# Hormone Therapy, Breast Cancer Risk and the Collaborative Group on Hormonal Factors in Breast Cancer Article

**DOI:** 10.1055/s-0040-1712941

**Published:** 2020-05

**Authors:** Luciano de Melo Pompei, César Eduardo Fernandes

**Affiliations:** 1 Faculdade de Medicina do ABC, Santo André, SP, Brazil; 2 Associação Brasileira de Climatério, São Paulo, SP, Brazil; 3 Federação Brasileira das Associações de Ginecologia e Obstetrícia, São Paulo, SP, Brazil

In August 2019, the safety of menopausal hormone therapy (MHT) returned to the scene again. Once more, its association with breast cancer risk came up, this time in an article published in the prestigious The Lancet (online in August and printed in September/2019). The subject is not new, and authors of the article are not newcomers either. By representing the Collaborative Group on Hormonal Factors in Breast Cancer, they brought a reanalysis of data from studies on the subject published so far.^1^

In summary, they evaluated data from 58 studies, out of which 24 were prospective and 34 were retrospective studies, covering almost 144 thousand postmenopausal women with breast cancer (cases) and close to 425 thousand without the disease (controls). A higher risk for developing breast cancer was found among MHT users. Women taking combined MHT for 1 to 4 years had a relative risk (RR) of 1.60 with a 95% confidence interval (CI) from 1.52 to 1.69, and for estrogen alone, it was observed a RR of 1.17 (95%CI: 1.10–1.26). For more prolonged use (5 to 14 years), RRs were 2.08 and 1.33, respectively.^1^

The first important point when evaluating this study is the fact that it is a reanalysis of data from other studies. In other words, data from other 58 studies were obtained and compiled, a total database was generated, and then analyzed. This was not a meta-analysis or systematic review, neither were data obtained from a same population at the same time or using the same method.

Soon after publication, the International Menopause Society (IMS) came public with their interpretation^2^ and found an echo in the Associação Brasileira de Climatério (SOBRAC, in the Portuguese acronym), which endorsed the IMS position and translated it into Portuguese under authorization.^3^

In their comments, the IMS mentioned that much information brought in the recent article is not new.^2^ ^3^ In fact, the previous publication of the Collaborative Group on Hormonal Factors in Breast Cancer itself, dating from 1997 and covering 51 studies brought similar data, with a RR of 1.35 for breast cancer associated with MHT use for ≥ 5 years.^4^

The Million Women Study also found similar values. The RR for breast cancer in MHT users was 1.66, in combined MHT users it was 2.00, and in users of estrogen MHT alone the RR was 1.30.^5^

What differs in the study published in The Lancet in 2019 regards the use of estrogen alone, because in this study, the use of estrogen alone presented higher risk, as well as the observational studies previously mentioned. However, in the Women's Health Initiative (WHI), the only large randomized study that assessed MHT effects on the risk for breast cancer, this effect was not observed. In the WHI, the RR for invasive breast cancer in users of estrogen MHT alone compared with placebo was 0.80 (95%CI: 0.62–1.04).^6^ In time, the WHI revealed that estrogen plus progestogen MHT increased the risk, but to a lesser extent than that reported by the Collaborative Group last August.^7^ Note that data from no randomized study, not even from the WHI, were included in this 2019 reanalysis of the Collaborative Group.

On the other hand, although this article did not bring much novelty in terms of MHT-associated breast cancer risk, there is a problem that may seem more relevant from the IMS perspective: most of the MHT assessed does not represent the most common current practices for this type of therapy. This is a result of the fact that in the Collaborative Group reanalysis, the median year of diagnosis of breast cancer cases for the entire database was 2002, whereas for retrospective studies, the median was year 1995. For American prospective studies, the median of the year of breast cancer diagnosis was 1999, and for Europeans it was 2007. Furthermore, in prospective studies, the average time of using MHT was 10 years in users diagnosed while using the therapy, and 7 years in former users.^1^ Therefore, much of the exposure to MHT preceded the first publication of the WHI study, after which the prescription practices changed substantially.^2^ ^3^

Another relevant criticism of this reanalysis involves the context of women who started MHT before the age of 45. In the presentation of results, the effect of MHT in this context was compared with women in the same age group that were not using MHT despite ovarian failure before the usual age, which led to the conclusion of a higher risk for breast cancer associated with MHT in these younger women. The correct thing would have been to compare the effects of MHT in women in the same age group while still in premenopause, because the most common in this age group is women still having ovarian hormone production and not the other way around.^2^ ^3^

However, the study in question has brought an important warning: obesity and overweight are associated with a higher risk for breast cancer, and this risk does not differ substantially from the risk associated with estrogen MHT alone in this same publication.^1^ In this scenario, attention should be paid to the high prevalence of female overweight and obesity that was also reported in Brazil.^8^

Finally, it is important to analyze the global effects of MHT and not just the effects on risk for breast cancer. The decision on whether or not to use this therapy should not fall solely on this issue. As an example, it is sufficient to retrieve information from the WHI study itself. Although it revealed a higher risk for breast cancer associated with MHT, neither a higher overall risk for all types of cancer together nor for mortality from these cancers were observed.^9^

The postintervention WHI data with 18 years of follow-up of participants showed no increase in the mortality rate from cancer in general or from cardiovascular causes.^10^

In conclusion, the publication of the Collaborative Group in The Lancet (2019) did not bring significant novelty regarding the risk for breast cancer in MHT users. Moreover, its results possibly do not reflect directly the real effects of the most current practices in MHT. In spite of all this, the decision to use MHT or not should always be made individually by taking into account the characteristics of each patient, the indications and absence of contraindications, the time since menopause (window of opportunity concept), the presence of comorbidities, as well as the values and opinions of the patient after due clarification.
